# Waterproofing in Arabidopsis: Following Phenolics and Lipids *In situ* by Confocal Raman Microscopy

**DOI:** 10.3389/fchem.2016.00010

**Published:** 2016-02-29

**Authors:** Batirtze Prats Mateu, Marie Theres Hauser, Antonio Heredia, Notburga Gierlinger

**Affiliations:** ^1^Department of Material Sciences and Process Engineering, University of Natural Resources and Life SciencesVienna, Austria; ^2^Department of Applied Genetics and Cell Biology, University of Natural Resources and Life SciencesVienna, Austria; ^3^Department of Molecular Biology and Biochemistry, University of MalagaMalaga, Spain; ^4^Institute for Building Materials, Eidgenössische Technische Hochschule ZürichZürich, Switzerland; ^5^Applied Wood Research Laboratory, Empa-Swiss Federal Laboratories for Material Testing and ResearchDübendorf, Switzerland

**Keywords:** lignin, cutin, wax, Raman, cuticle, secondary cell wall, trichomes

## Abstract

Waterproofing of the aerial organs of plants imposed a big evolutionary step during the colonization of the terrestrial environment. The main plant polymers responsible of water repelling are lipids and lignin, which play also important roles in the protection against biotic/abiotic stresses, regulation of flux of gases and solutes, and mechanical stability against negative pressure, among others. While the lipids, non-polymerized cuticular waxes together with the polymerized cutin, protect the outer surface, lignin is confined to the secondary cell wall within mechanical important tissues. In the present work a micro cross-section of the stem of *Arabidopsis thaliana* was used to track *in situ* the distribution of these non-carbohydrate polymers by Confocal Raman Microscopy. Raman hyperspectral imaging gives a molecular fingerprint of the native waterproofing tissues and cells with diffraction limited spatial resolution (~300 nm) at relatively high speed and without any tedious sample preparation. Lipids and lignified tissues as well as their effect on water content was directly visualized by integrating the 1299, 1600, and 3400 cm^−1^ band, respectively. For detailed insights into compositional changes of these polymers vertex component analysis was performed on selected sample positions. Changes have been elucidated in the composition of lignin within the lignified tissues and between interfascicular fibers and xylem vessels. Hydrophobizing changes were revealed from the epidermal layer to the cuticle as well as a change in the aromatic composition within the cuticle of trichomes. To verify Raman signatures of different waterproofing polymers additionally Raman spectra of the cuticle and cutin monomer from tomato (*Solanum lycopersicum*) as well as aromatic model polymers (milled wood lignin and dehydrogenation polymer of coniferyl alcohol) and phenolic acids were acquired.

## Introduction

*Arabidopsis thaliana* is the model organism for plant genetics and biology due to, among other reasons, the small genome size, its short generation time, the knowledge of its whole genomic sequence, the large amount of genetic resources (i.e., mutants), and simple genetic transformation and cultivation protocols (Kaul et al., 2000). Arabidopsis has been extensively used to reveal the genetic basis and study the different plant polymers e.g., cellulose, hemicellulose, pectin, lignin, cutin, wax, and suberin (Beisson et al., 2012; Atmodjo et al., 2013; Pauly et al., 2013; McFarlane et al., 2014; Barros et al., 2015). The non-polysaccharide polymers, lignin, cutin, and suberin played an evolutionary important role during the transition from water to land by conferring waterproofing properties and mechanical strength. The need for mechanical support and the intensified water demand due to plant architectural changes such as branching and increasing height triggered the occurrence of the aforementioned polymers (Langdale and Harrison, 2008).

Lignin is a branched heterogeneous phenolic polymer originating from the oxidation of precursors called monolignols (p-coumaryl, coniferyl, and sinapyl alcohols) and subsequent radical coupling (Boerjan et al., 2003). It is known to act as an embedding media (together with the hemicelluloses) for the cellulose fibrils during secondary cell wall formation (Carpita et al., 1993). The hierarchical nature of biomaterials—the optimization at different length scales (levels of hierarchy)—is responsible for the achieved macroscopic properties (Bergander and Salmen, 2000). The reinforced bio-composite (Fratzl et al., 2004) has elevated resistance to compression failure and superior hardness (Gindl et al., 2004; de Borst et al., 2012; Burgert and Keplinger, 2013). Lignin stiffens the cell wall (Jones et al., 2001) and confers waterproofness and resistance to the negative pressure generated during water transport in the xylem as well as to biotic and abiotic stresses (Sarkanen and Ludwig, 1971). Arabidopsis has been used to study the secondary xylem development as model for wood formation (Chaffey et al., 2002) and lignification (Dima et al., 2015).

Cutin and wax form a lipid barrier called cuticle, which covers all aerial parts of land plants. The cuticle appeared in early plants around 450 million years ago and has been preserved because of its essential role during the colonization of land (Edwards, 1993) challenging gravity, desiccation, and brusque changes in temperature (Waters, 2003). The cuticle has further functions including abiotic and biotic stress protection (e.g., against pathogens or insects), the regulation of the flux of water, gases, and solutes and the sealing of aerial organs of leaves, fruits, petals, and non-lignified stems of the outer plant cell wall (Kerstiens, 1996; Pollard et al., 2008). It offers also mechanical rigidity to the plant (Dominguez et al., 2011). It consists of intracuticular waxes embedded in cutin and an epicuticular layer of crystalline wax at the outer part. In minor amounts it also includes triterpenoids, phenolic compounds including cinnamic acid, flavonoids, and secondary metabolites (Hunt and Baker, 1980). This wax is composed mainly of long chain aliphatic molecules (alkanes) derived from long chain fatty acids, and alcohols. Cutin can be described as a polyester matrix (mainly primary ester bonds) of hydroxy fatty acids and hydroxy-epoxy fatty acids C16 and C18 (Heredia, 2003; Pollard et al., 2008). The cuticle is a variable membrane depending on the function and necessity and environmental conditions (Macherius et al., 2011; Domínguez et al., 2015).

Confocal Raman Microscopy (CRM) has shown a high potential for *in situ* chemical characterization of plants due to its non-destructive nature (Agarwal, 2006; Gierlinger and Schwanninger, 2006, 2007; Gierlinger et al., 2012). The inelastic Raman scattering, recorded as an energy shift by the CCD camera, reflects the molecular vibrations (e.g., bond stretching, rotation, torsion) of the sample and thus the nature of its components (Mueller et al., 2003). The advantage of CRM is that position-resolved molecular fingerprints can be generated with a lateral resolution of 300 nm and a z resolution of ~700 nm (532 nm, NA 1.4). A Raman image is composed of thousands of spectra in which each local position carries its own chemical information and each spectral position has its own molecular identity (Smith and Dent, 2005). The large amount of data generated by Raman imaging can be overcome with the use of multivariate methods that help in the management and interpretation of the data. A blind source of unmixing is helpful since a spectrum can be comprised of many overlapping bands in which the human-eye assignment can be tedious and in many cases impossible. Several multivariate methods have been applied in Chemometrics (Geladi et al., 2010) and also on Raman images of plant cell walls (Gierlinger, 2014). Vertex Component Analysis (VCA) is an iterative method that finds the most pure spectra on the sample by projecting the endmembers in an orthogonal space (the endmembers are independent of each other; Nascimento and Dias, 2005). VCA is a fast, powerful, and reliable algorithm that is able to detect and extrapolate the hypothetical end members using the raw data without any need of dimensionality reduction.

In this work, CRM was used to generate Raman images on a stem microsection of Arabidopsis. The hyperspectral data were analyzed by a univariate method (band integration) and VCA to reveal new insights into the topochemistry of the plant polymers. The correspondent average and endmembers spectra were extracted for a detailed analysis and compared with reference spectra of non-polysaccharide plant polymers.

## Materials and methods

### Plant material and micro-sectioning

The stem of a 3 week old Arabidopsis (accession Columbia) was transversally cut above ground/the rosette leaves for embedding in polyethylenglycol (PEG) 2000 (Sigma, Austria) following the protocol described in Gierlinger et al. (2012). The embedded blocks were cut in 2 μm thick cross sections using a rotary microtome (RM2235, Leica Biosystems Nussloch GmbH, Germany). The sections were washed with distilled water to remove the PEG. For Raman imaging single sections were put on a glass slide with a drop of water, covered with a coverslip (0.17 mm thick) and sealed with nail polish to avoid water evaporation.

Cuticles from green tomato (*Solanum lycopersicum* L., var. *Casacada*) fruits were isolated by incubation for 5 days in a 2% pectinase/0.2% cellulose solution at pH = 3.6 and treated with chloroform:methanol (3/1, v/v) for 3 h to remove waxes. Dewaxed cuticles were depolymerized by saponification in 1% KOH/methanol for 6 h under refluxing conditions. The filtrate was neutralized with HCl 1N and extracted with diethyl ether. The solid contains about 82% (w/w) of 10(9), 16-dihydroxyhexadecanoic acids and minor amounts of other hydroxyacids and phenolics as *p*-coumaric acid (Heredia-Guerrero et al., 2014). This mixture will be considered as cutin monomer sample in the present work.

To help in band assignment of lignin structures Raman spectra were acquired from milled wood lignin (MWL) of spruce and beech. MWL is a preparation procedure (extraction with a dioxane–water mixture) widely used for structure studies of lignin and was compared to an artificial lignin dehydrogenation polymer (DHP) preparation based on coniferyl alcohol treated with H_2_O_2_ in the presence of the enzyme peroxidase. All lignin preparations have been provided by the group of Antje Potthast (University of Natural Resources and Life Sciences, BOKU, Vienna).

### Confocal Raman microscopy and data analysis

Raman spectra from the native Arabidopsis cross sections were acquired using a confocal Raman microscope (alpha300RA, WITec GmbH, Germany) with a 100x oil immersion objective (NA 1.4, 0.17 mm coverslip correction; Carl Zeiss, Germany). The sample was excited with a linear polarized (0°) coherent compass sapphire green laser λ_ex_ = 532 nm (WITec, Germany). The scattered Raman signal was detected with an optic multifiber (50 μm diameter) directed to a spectrometer UHTS 300 (WITec, Germany; 600 g mm^−1^ grating) and finally to the CCD camera DU401A BV (Andor, Belfast, North Ireland). The Control Four (WITec) acquisition software was used for the Raman imaging set up. The laser power was set at 30 mW and a short integration time between 0.1 and 0.5 s was chosen in order to ensure fast mapping and avoid changes in the components structure. One spectrum was taken every 0.3 μm to reach the maximum possible diffraction limited spatial resolution (*r* = 0.6 × λ/NA).

Single Raman spectra of the cutin monomer and the milled wood reference samples were performed with a 100x objective (NA 0.9) and the λ_ex_ = 785 nm laser (optic multifiber with a 100 μm diameter) using an integration time of 1 s and 20 accumulations. The grating used was the same (600 gmm^−1^) and the CCD camera corresponded to the model DU401A BR DD (Andor, Belfast, North Ireland). For the isolated tomato fruit cuticle the same instrument configuration was used and stack scans (x–y–z) performed with a z step size of Δz = 0.350 nm. Average spectra of the total scanned area were extracted and averaged for the first 2 μm from the outer cuticle surface (including the very thick epicuticular wax layer).

Cosmic ray removal was carried out before any further analysis in all cases using the WITec Project Plus 4.0 software (WITec, Germany). Raman images of the cross-sections of Arabidopsis were generated with the same software by using a sum spectral filter which allows specific band integration. This provides chemical images based on the specific bands attributed to different plant components e.g., lignin, water, and cutin plus waxes. Furthermore, based on the integration images, average spectra were extracted by setting an intensity threshold in order to obtain the characteristic Raman signature of the cuticle and the lignified tissues, separately.

Detailed analysis on lignified tissues and cuticles of Arabidopsis were done by Vertex Component Analysis (VCA) in Cytospec (v.2.00.01) using different number of endmembers and areas within the spectral region of 300–3050 cm^−1^. All calculated endmember spectra were exported into OPUS 7.0 (Bruker, Germany) for direct comparison. All the reference spectra (including milled wood lignins, cutin monomer, tomato cuticle, ferulic, and coumaric acid) were min–max normalized on the aromatic band at 1600 cm^−1^ for better comparison.

## Results

### Waterproofing polymers at the micron-level

**Figure 1:**
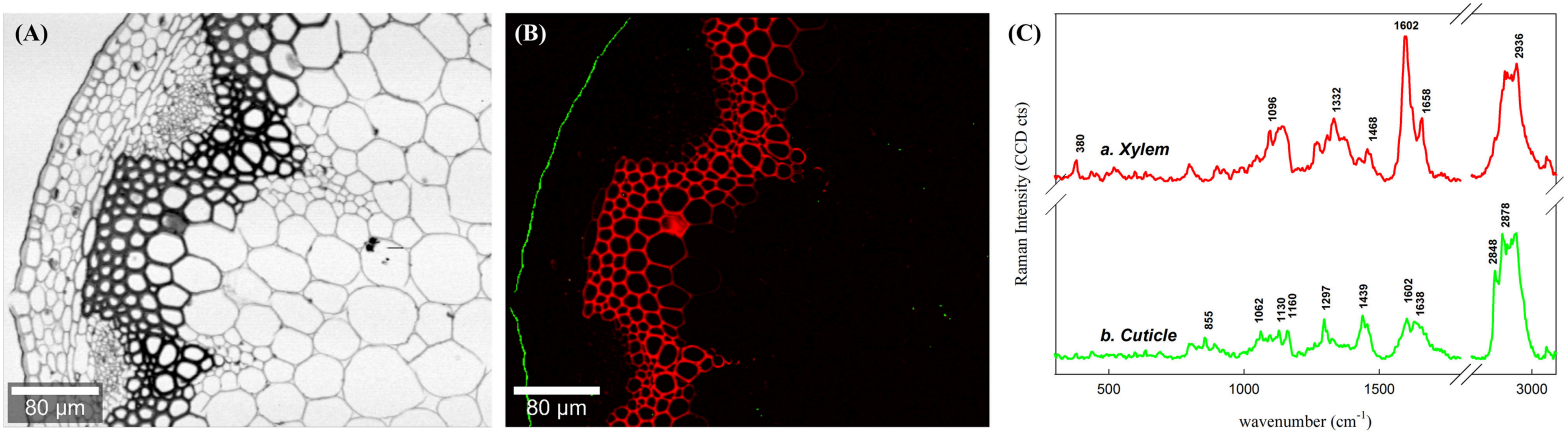
**Raman images of a cross section of an Arabidopsis stem by univariate data analysis**. Figure captions correspond to images generated by integrating over the **(A)** water main band at around 3400 cm^−1^ and **(B)** lignin around the spectral area 1550–1700 cm^−1^ (in red) together with lipids (in green) at 1299 cm^−1^. **(C)** Average spectra of calculated by setting an intensity thresold over the image **(B)** for lignin and cuticle in the stem, separetely. Note that the minimal water content in the section matches the areas covered by lignin and cuticle.

To present an overview of the distribution of waterproofing polymers in the Arabidopsis stem Raman images were first calculated through integration over specific marker bands (Figures 1A,B). Integration over the broad water band around 3400 cm^−1^ visualizes directly the distribution of water within the investigated microsection (Figure 1A). High water content is represented by the white areas, whereas the dark areas point to low water content and thus impregnation of waterproofing polymers. The lumina of the cells appear white as they are almost completely water filled, except a few dark deposits are found. The parenchyma cell walls are colored in gray representing a high water content within the thin cell walls. On the contrary the thick walled sclerenchyma and xylem cells are almost black and thus represent low water content. Furthermore, a very thin black layer, the cuticle, is visualized at the outer waterproof surface (Figure 1A). The visualized hydrophobicity is due to the impregnation of non-polysaccharide polymers as proofed by integrating the marker bands of lignin (1550–1700 cm^−1^) and cuticular lipids (1297 cm^−1^, green), respectively. The red coloration represents impregnation with lignin and is restricted to the xylem and sclerenchyma cells, whereas cuticular lipids (waxes and cutin; green color) are confined to the epidermal outer cell wall surface, the cuticle (Figure 1B).

By setting an intensity threshold on the two different integration images, average spectra of the cuticle and the lignified tissues were extracted individually and compared (Figure 1C). The average spectrum of the lignified tissues (Figure 1Ca) is a combination of cellulose (bands at 380 and 1096 cm^−1^, (Wiley and Atalla, 1987a,b), probably hemicelluloses (although the bands are not distinguishable at first sight) and lignin with typical bands at 1602 and 1658 cm^−1^ (see assignments in Table 1; Agarwal, 1999). The average spectrum of the outer cuticle (Figure 1Cb) has also a high intensity in the CH stretching region (~2800 cm^−1^) due to the abundance of methylene CH2 in both aliphatic waxes and cutin, almost no carbohydrate contribution, but a small characteristic band of pectin at (855 cm^−1^). The cuticle spectrum is characterized by clear bands at 1439, 1297, and 1602 cm^−1^.

**Table 1:** **Assignments of the reference spectra summarized in Figure 2**.

**Wavenumber**	**Assignment (Literature)**	**Lignin and phenolic acids**					**Cuticle**	
		**DHP**	**MWL Spruce**	**MWL Beech**	**p-Coumaric acid**	**Ferulic acid**	**Cutin monomer**	**Tomato cuticle**
370	Lignin of sugarcane pith (Agarwal unpublished cited in [1]). Syringyl unit in hardwoods [18]	370	370	371				
531	Skeletal deformation [1]			531				
	Lignin of sugarcane pith (Agarwal unpublished cited in [1])							
815	Out of plane bending C–OH [16]	817				814		814
833					835			
850–862	α-Glycosidic bond in pectin [16]						856	866
	Skeletal vibration pyranoid ring				864			
1033	C–O of aryl-O–CH3 and aryl-OH [2]			1039				
1037	Heavy atom (CC and CO) stretching [11]							
1061	ν C–C in cuticular wax [3, 4, 6, 15]						1063	
1136	Coniferyl/Sinapilaldehyde [1]	1134						
1170–1178	Ring δ ip (CH), ν(C–O–C) ester [3, 17]				1177	1177	1172	1167
	ν (C–O) [10]							
1266	δ ip (= C-H) *cis* of lipids [7, 8]		1272		1260	1272		
1272	Aryl-O stretching of aryl-OH and aryl-O–CH3 (G unit) [1]							
1274	ν(C–C) [10]							
1295	δ (CH2) twisting saturated wax [9]						1299	1298
1303	τ (CH2) lipids [4]							1307
1331–1334	Aliphatic O–H bend [1]	1331		1330				
	Syringyl lignin [5]							
1434	δ CH3 asym. sym. [10]					1432		
1441	δ (CH2) Lipids [7]						1443	1438
1453	CH3 bending in OCH3 [1, 2]	1453	1453	1463				
1588	Aromatic ν (C–C) phenolic compound [4]						1590 sh	
1607	Aromatic ν (C = C) Phenolic compound [3] e.g., Lignin [2]	1602	1599	1601	1605	1601	1605	1604
1621	ν C = C of coniferylaldehyde/sinapaldehyde [1]			1621				
1632	Unsaturated ν C = C of phenolic compound [3]				1636	1628	1631	1632
	ν C = C of coniferyl aldehyde [1, 12, 13, 14]							
1657–1660	C = O coniferyl aldehyde [1], C = C coniferyl alcohol [12]	1652	1667	1667				1662
1640–1680	Sym. ν C = O of carboxylic acid dimer							1682
1720	ν (C = O) cuticular wax [4]							1727

*All values are given in wavenumbers (cm^−1^). ip, in plane; δ, deformation; τ, torsion; ν, stretching; sh, shoulder; sym, symmetric; asym, asymmetric. [1], (Agarwal et al., 2011); [2], (Agarwal and Ralph, 1997); [3], (Prinsloo et al., 2004); [4], (Trebolazabala et al., 2013); [5], (Sun et al., 2012); [6], (Yu et al., 2008); [7], (Schulz and Baranska, 2007); [8], (Da Silva et al., 2008); [9], (Edwards and Falk, 1997); [10], (Ram et al., 2003); [11], (Wiley and Atalla, 1987a); [12], (Agarwal and Ralph, 2008); [13], (Stewart et al., 1997); [14], (Hanninen et al., 2011); [15], (Wu et al., 2011); [16], (Synytsya et al., 2003); [17], (Heredia-Guerrero et al., 2014); [18], (Agarwal et al., 2015)*.

**Figure 2:**
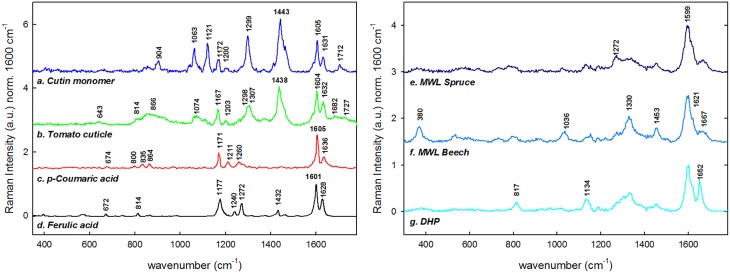
**Reference Raman spectra of lipids and phenolics**. The spectra were cut (300–1800 cm^−1^), base line corrected and normalized over the main aromatic stretching band at 1600 cm^−1^. **(a)** Cutin monomer, **(b)** average spectra of the outer part of the tomato cuticle, **(c)** p-coumaric acid, **(d)** ferulic acid, **(e)** milled wood lignin from Spruce (*Picea abies*), **(f)** milled wood lignin of Beech (*Fagus sylvatica*), and **(g)** synthethized dehydrogenation polymer of lignin (DHP). The spectra are base line corrected and normalized over the 1600 cm^−1^ band.

Figure 2 summarizes the reference spectra used in this study for comparison. Spectra of a cutin monomer mixture (Figure 2a), isolated green tomato cuticle (Figure 2b), and p-coumaric acid (Figure 2c) and ferulic acid (Figure 2d) were used for comparison with the stem cuticle of Arabidopsis. Cutin monomer mixture is composed of non-esterified polyhydroxy fatty acids and low amount of phenolics, whereas the outer part of the native tomato cuticle used for the reference measurements is mainly constituted of waxes. If the two spectra are compared the two sharp bands at 1063 and 1121 cm^−1^ are only found in the cutin monomer, whereas bands around 1170, 1299, and 1440 cm^−1^ are found in both samples. The bands characteristic for p-coumaric acid at 1605, 1636, and 1171 cm^−1^ are found in the cutin monomer mixture as well as in the tomato cuticle. Additionally, spectra of milled wood lignin (MWL) of softwood (spruce, Figure 2e) and hardwood (beech, Figure 2f) and of the dehydrogenation polymer of coniferyl alcohol (DHP; Figure 2g) are shown: having all together the typical strong aromatic ring stretching band around 1600 cm^−1^. The band typical for guaicyl units at 1272 cm^−1^ is seen more pronounced in MWL of spruce, whereas the typical syringyl unit bands at 370, 1036 and 1330, and 1453 cm^−1^ are only clearly seen in MWL of beech. In DHP the bands at 1134 and 1652 cm^−1^ are more pronounced than in MWL. All characteristic band positions and possible assignments are resumed in Table 1.

### Phenolic barriers: Lignin in cell wall, cell corners, and compound middle lamella

**Figure 3:**
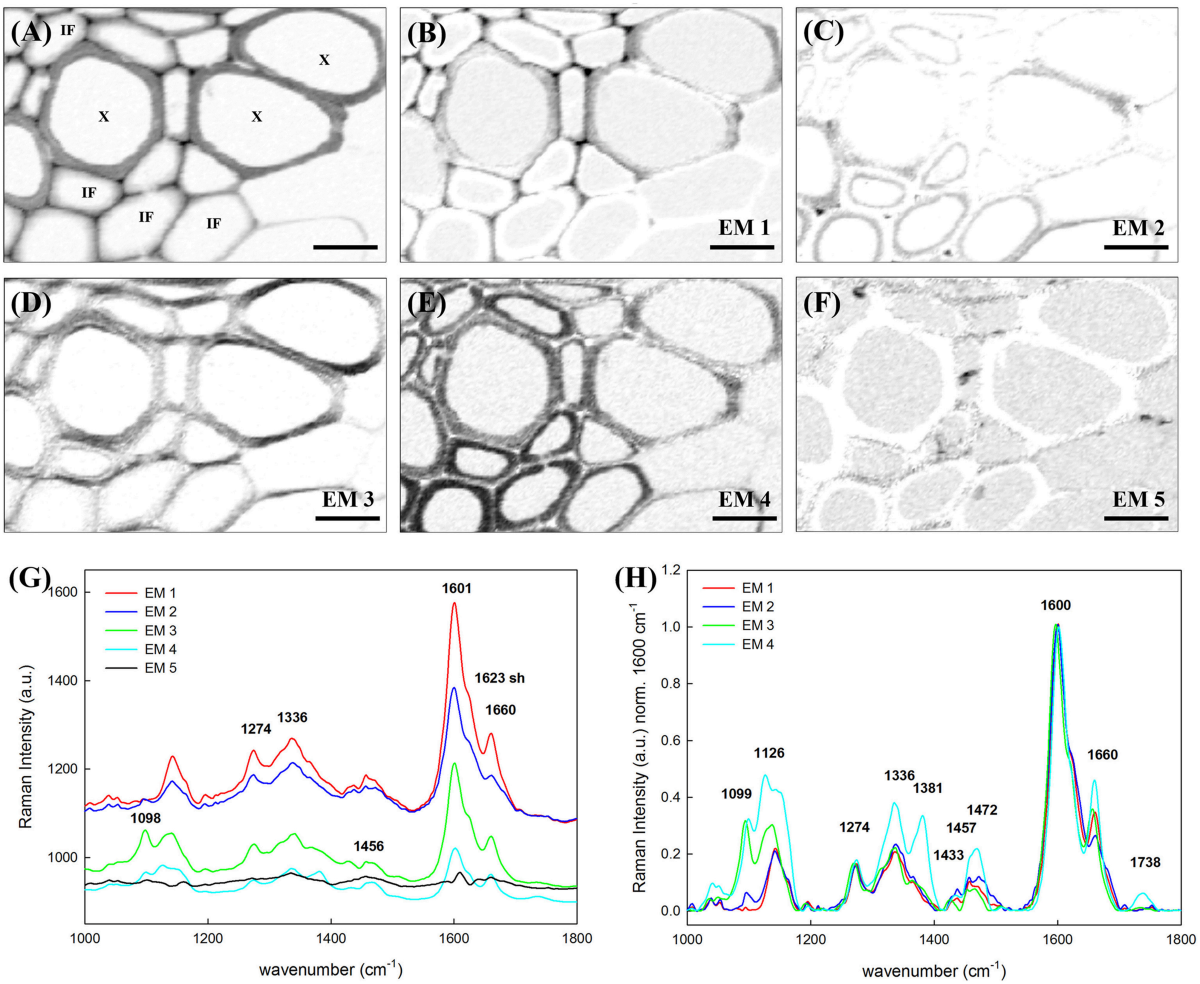
**Vertex component analysis of lignified tissue within an Arabidopsis stem**. Scale bar: 8 μm. **(A)** Image generated by integrating over the lignin main band at 1600 cm^−1^. The X is pointing to the vessel elements in xylem. IF stands for interfascicular fiber. **(B–F)** Abundance maps of the endmembers generated by VCA (the intenisty profiles of abundance maps are scaled 0–1): **(B)** EM 1 depicts the cell corners and middle lamella together with the tangential walls of the vessel element, **(C)** EM 2 points to the inner cell wall of the IFs whereas **(D)** EM 3 highlights mostly the radial cell walls of the vessel. **(E)** EM 4 is characteristic of the IFs cell wall and **(F)** EM 5 depicts a high angle (and parallel to laser polarization) orientation of the cellulose microfibrils respect to the longitudinal axis. **(G)** Endmembers corresponding to the abundance maps above. EM 1–4 endmember spectra were normalized over the lignin main band at 1600 cm^−1^ and plotted in **(H)**.

A selected area of the Arabidopsis scan comprising sclerenchyma fibers and xylem elements was analyzed in detail, using vertex component analysis (VCA) with a total of six endmembers and the spectral region 1000–1800 cm^−1^. In order to compare univariate vs. multivariate statistical approaches, Figure 3A shows first the false color image generated by integration over the main lignin band at 1600 cm^−1^ (univariate approach). The lignin amount is higher in cell corner and compound middle lamella indicated by the darker color. Furthermore, it can be seen that the cell walls of xylem vessels (X) contain more lignin (darker gray) than the cell walls of the interfascicular fibers (IF). Figures 3B–F display the abundance maps of the endmembers (EM) spectra shown in Figure 3G and after baseline correction and min–max normalization over the 1600 cm^−1^ band in Figure 3H (fifth EM not shown as no contribution at 1600 cm^−1^). The sixth EM corresponding to the lumen (water) is not shown. The EM 1 differentiates the upper cell corners near the vessel elements (X), middle lamella, and the tangential cell wall of the vessels. The correspondent EM 1 (red spectrum Figures 3G,H) shows the highest lignin band (1600 cm^−1^) and almost no carbohydrate contribution. The EM 2 depicts an inner layer toward the lumen and the cell corners between the IF (Figure 3C). The spectrum (in blue) shows lower lignin contribution at 1600 cm^−1^, a shoulder at 1623 cm^−1^ and the lowest intensity of the 1660 cm^−1^ peak relatively to the other EMs (Figure 3H), pointing to a change in lignin composition. The next abundance map in Figure 3D (EM 3) accentuates cell wall regions along the horizontal axis of the image, which points to an effect of the laser polarization direction. A high band at 1098 cm^−1^ in the EM spectrum (green) explains this effect due to an enhancement of this band in regions where the cellulose microfibril is aligned with a high angle with respect to the plant axis. Thus, a higher angle is observed in the vessel wall than in the IF, where the high intensity is restricted to a very small S1 layer. EM 4 (Figure 3E) highlights the cell wall of IFs and to less extent the vessel cell wall. This EM spectrum shows after normalization clearly the highest proportion of cellulose (Figure 3H, turquoise spectrum high at 1126, 1381 cm^−1^). Besides, the pronounced band at 1336 cm^−1^, which was seen as a marker band in beech MWL due to higher amount of incorporated syringyl units (Figures 2e,f), points to a higher incorporation of syringyl units in the secondary cell wall of the IF. The last EM (Figure 3F) pictures several deposits in the lumen and the interfaces between cells. The EM spectrum (black spectrum) shows a broad band at 1456 cm^−1^ and other characteristics for a mixture of lipids and proteins. To summarize up VCA is not only able to detect changes in lignin amount, but also to reveal topochemical changes in the heterogenous lignin polymer composition.

### Lipid barriers: Wax and cutin in the cuticle

**Figure 4:**
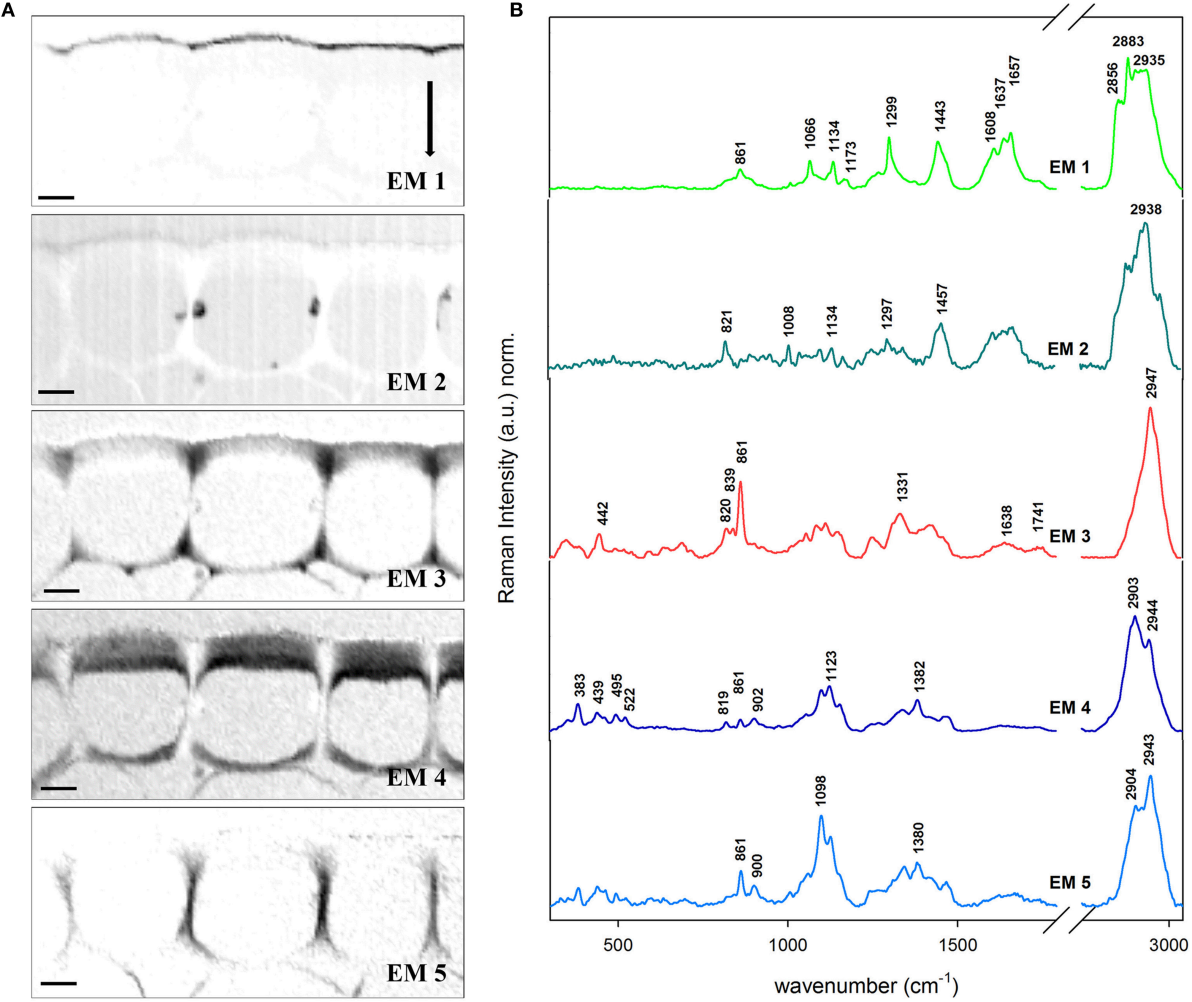
**Vertex component analysis on the cuticle of Arabidopsis stem**. Scale bar: 4 μm. The number of endmembers chosen for initialization was six. **(A)** Endmember's abundance maps (endmember 6 correponding to the background is not displayed). The intenisty profiles of abundance maps are scaled 0–1. **(B)** Endmember spectra corresponding to the abundance maps showed in **(A)**. EM 1 describes the cuticle and EM 2 deposits in the lumen and pectin near the cuticle. Contrarely, EM 3 is distinguising pectin accumulated in the epidermis while EM 4 is typical for cellulose parallel to the longitudinal axis. EM 5 is peculiar for high angle cellulose orientation. Each spectrum is baseline corrected and is scaled differently in order to facilitate the observation of minor bands. The arrow indicates the laser polarization direction.

Epidermis and cuticle of the Arabidopsis cross-section was also object of VCA with a total of six EMs. The abundance maps of EM 1–5 (EM 6 corresponded to background and represents just water and is therefore not shown) are displayed together with the related EM spectra (Figures 4A,B). The EM 1 abundance map depicts the cuticle (around 2 μm thick) which is characterized by the presence of pectin (855 cm^−1^), lipids (1299, 1441, and 2883 cm^−1^) including cuticular waxes (1066, 1299, and 1720 cm^−1^) and phenolic compounds (1608, 1637, and 1657 cm^−1^). EM 2 displays the cytoplasm in the lumen and partly the cuticle as well. The spectra again reveal the presence of pectin at 839 cm^−1^, proteins and lipids (1003 and 1457 cm^−1^) and phenolic compounds in the 1540–1670 cm^−1^ region. However, most of the pectin signal (839, 861, and unique band at 2947 cm^−1^) is observed in the cell corners of the epidermis with a triangular distribution (EM 3). Cellulose contribution is mainly restricted to the epidermis, and separated in EM 4 (tangential wall) and EM 5 (radial wall) due to high angle of the cellulose microfibrils with respect to the stem axis. The thick outer tangential wall displayed by EM 4 shows a gradient structure with higher cellulose intensity near the cytoplasm and less toward the cuticle, where the pectin (EM3) is more dominating. In EM 4 distinctive bands at lower wavenumber region appear which are attributed to both cellulose and hemicelluloses (383, 495, and 522 cm^−1^) and pectin (439 cm^−1^). In fact this data show that the cuticle is a modification of the outer cell wall of the epidermal cells.

### *Arabidopsis* dries its hair

**Figure 5:**
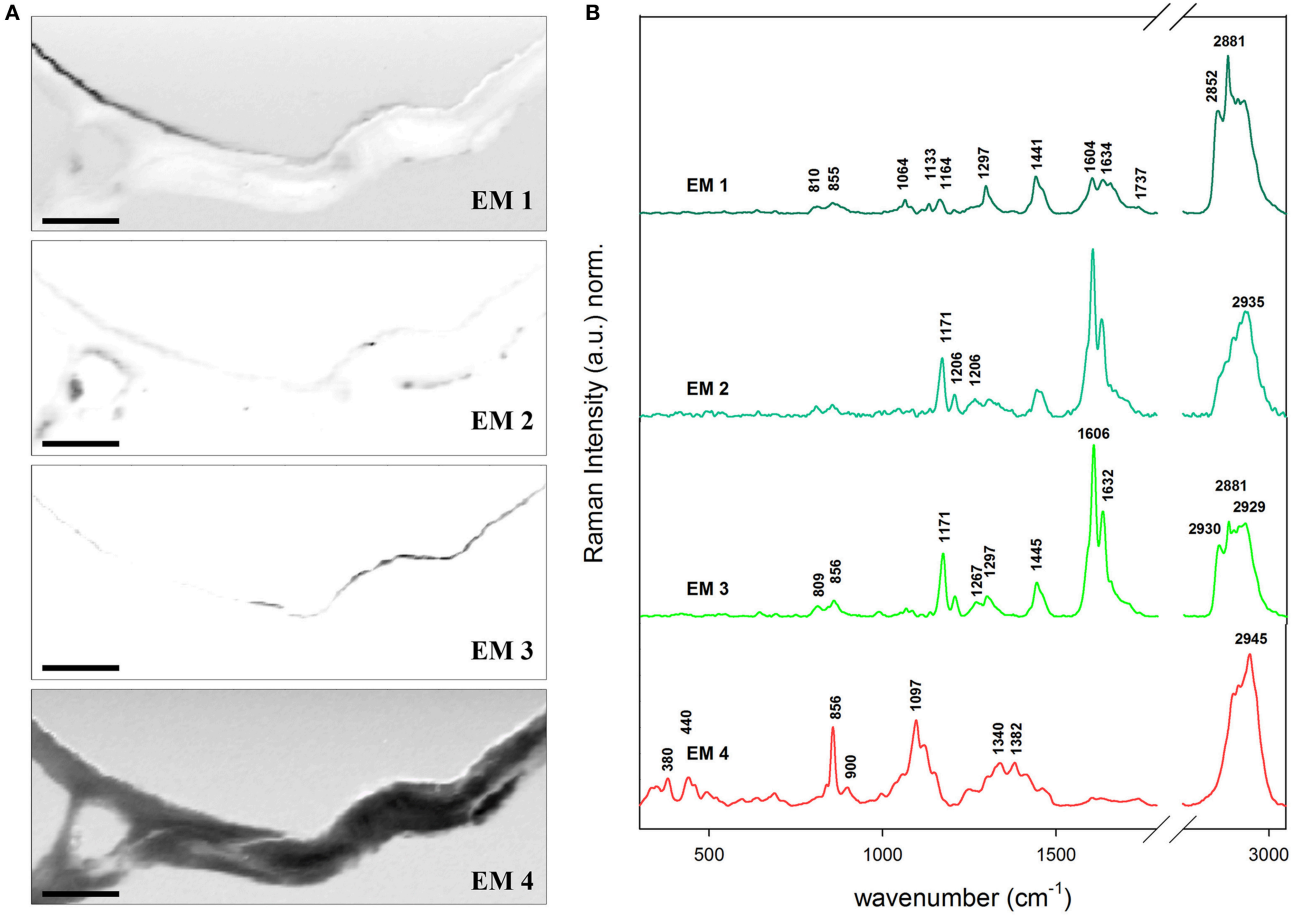
**Vertex component analysis on a trichome of Arabidopsis stem. (A)** The captions correspond to the abundance maps of the endmembers. The intensity profiles of the abundance maps are scaled 0–1. The initial rank chosen was five endmembers (background not shown) based on the main components in epidermis: cuticular wax (EM 1), cuticular wax rich in phenolic compounds e.g., lignin (EM 2 and 3) and polysacharides cellulose and pectin (EM 4). The transition from a cuticle poor in the stem to a rich in phenolic compounds in hair is observed between EM 1, and EM 2 and EM 3. **(B)** Endmember's characteristic spectra. Each spectrum is baseline corrected and is scaled differently in order to facilitate the observation of minor bands. Scale bar 10 μm.

Additionally a non-branched trichome of the stem of *Arabidopsis* was analyzed in detail by Confocal Raman Microscopy and VCA (Figure 5). The transition between a lipid-rich (near the stem) to a high-phenolic-containing cuticle (in the outermost extreme of the hair) is observed: the abundance maps of EM 1, EM 2, and EM 3 depict clearly three kinds of cuticle in regard of the phenolic components (1170 and 1560–1700 cm^−1^) and the lipidic and cuticular wax content (1297 cm^−1^). The polysaccharide polymers are gathered in EM 4 which shows peaks for pectin (856 cm^−1^), cellulose (380 and 1097 cm^−1^), and hemicelluloses (bands at lower region of the spectrum). EM 1 has the characteristic peaks for cuticular lipids. EM 3 as well but additionally higher intensities at the positions 1171 (ring CH deformation), 1606 and 1632 cm^−1^ (ring C=C and C=O stretching) typical for p-coumaric acid (see spectrum c of Figure 2). On the contrary, the band at 2881 cm^−1^ (anti-symmetric CH2 stretching of lipids and proteins) is higher in EM 1 when compared to EM 3 meaning that the relative lipid content in the hair cuticle (EM 3) is lower than in the stem (EM 1). The EM 2 represents several deposits in the cytoplasm of the epidermal cell on the left side of the image and contributes slightly to the cuticle. The size of the lipid chains at these positions drops since the bands in the endmember spectra accounting for the CH2 stretching decrease tremendously, while the phenolic content is also similar high like in the before described hair cuticle. Actually, the band at 2935 cm^−1^ that is characteristic of the symmetric CH3 stretching of lipids but also of the O–CH3 stretching in lignin increases in relation to the others. VCA reveals that for hydrophobizing the stem cuticle and the trichome cuticle different strategies have evolved.

## Discussion

It is amazing to observe how plants have evolved to overcome “most” of their enemies and to optimize their living conditions even under dry conditions. Most of the physiological functions of the cuticle are a consequence of the physical and chemical properties of cutin, its interaction with the cell wall components and the subtle regulation of other minor compounds. These key physical properties, belonging to three basic areas of physics—thermodynamics, hydrodynamics, and mechanics—are not isolated but they largely influence each other's performance. The design requirements of each property can be incompatible with the properties needed for the others to maximize their functions, leading to a necessary compromise (Dominguez et al., 2011). Thus, the biological role of waterproofing polymers is clear since plants have to keep their feet wet and avoid simultaneously plasmolysis due to out-of-equilibrium medium as well as to keep clean and be protected against new enemies. The characterization of waterproofing lipid and phenolic barriers is of great interest in plant science due to the mentioned biological relevance but also for their use as feedstock for long chain components and biodiesel production (Tsubaki and Azuma, 2013). Lately, different approaches in genetic engineering have been proposed to increase the yield of wax esters in tobacco plants (Aslan et al., 2015). In the same way, lignin has been recognized for its potential as renewable raw material for producing alkylated phenolic compounds, fuels and renewable chemicals (Kleinert and Barth, 2008; Bugg and Rahmanpour, 2015).

### Imaging waterproofing polymers and also their effect on water content

The ability of *in situ* clustering and detecting polymers involved in a process such as waterproofing of the plant is essential to know more about the responsible mechanisms behind (Altartouri and Geitmann, 2015). In this study the topochemical distributions of non-polysaccharide polymers and water content were elucidated with a resolution of about 300 nm based on Raman imaging using univariate as well as advanced multivariate data analysis. The univariate analysis of the 2D Raman hyperspectral data revealed an immediate overview about changes in chemical composition between the main structural parts of the plant stem (Agarwal and Atalla, 1986; Agarwal, 2006; Gierlinger and Schwanninger, 2006, 2007) (Figure 1B): the cuticle was separated by integrating over the main lipid band around 1300 cm^−1^ (Wu et al., 2011), and the xylem was highlighted by integrating over the aromatic lignin band from 1540 to 1700 cm^−1^ (Agarwal and Ralph, 1997; Prats-Mateu et al., 2014). The water image (Figure 1A) showed directly the effect of the waterproofing polymers by an opposite hand picture with low water content in the regions where the non-polysaccharide polymers have been detected.

Representative average spectra of the lignified tissues and cuticle could be extracted through integration images and set intensity thresholds (Figure 1C). The spectrum of the lignified tissue (a) is a combination of cellulose (bands at 380 and 1096, Wiley and Atalla, 1987a,b, 2936 cm^−1^), hemicelluloses (not distinguishable from the first), and lignin with bands at 1270, 1332, 1602, 1658 (see assignments in Table 1), and 2936 cm^−1^ due to the antisymmetric C–H stretching in O–CH3 (Agarwal, 1999). The absence of pectin at position 855 cm^−1^ (α-1,4-glycosidic bond; Synytsya et al., 2003) and the presence of lignin together with a thickening of the cell wall in these lignified tissues is an indication of a mature developmental stage of the tracheary elements (vessels and fibers) and their (ongoing) dead stage (Bollhöner et al., 2012). The cuticle by contrary was strongly defined by the presence of lipid components and slightly pectin (855 cm^−1^) due to the close vicinity of the pectin rich epidermis. The lipidic part is defined mainly by an increase in the C–H and C–C groups due the large aliphatic chains characteristics of waxes and hydroxy fatty acids from cutin. This is visible in the spectrum at the positions 1062, 1297, 1441, and 2848 (symmetric) and 2878 cm^−1^ (asymmetric CH2 stretching vibration) (Weissflog et al., 2010; Littlejohn et al., 2015). The last two bands can be used to determine the crystallinity of the cuticle (Ho and Pemberton, 1998; Greene and Bain, 2005; Littlejohn et al., 2015) which differs in its inner (intracuticular wax) and outer parts (epicuticular wax; Heredia-Guerrero et al., 2014).

The univariate methods i.e., band integration is very suitable for bands, which are not (or only slightly overlapping) with others, to give a fast overview. Nevertheless, this approach is powerless regarding the splitting of the pure components or to separate changes in polymer amount from changes in polymer composition (side chains, crystallinity), which might go hand in hand. Furthermore, due to the multicomponent nature of biological materials we almost always have to deal with overlapping bands, which can lead to misleading conclusions or hide important changes.

### Necessity of multivariate methods to track subtle changes in lignin composition

Lignin is a heterogeneous phenolic compound which complexity is still puzzling scientist around the world (Voxeur et al., 2015). The diversity of the monomer (oligomer) coupling and polymerization mechanisms makes the study of this polymer a difficult task (Boerjan et al., 2003). The breaking pathways of lignin (Mar et al., 2015) are of great interest since it has been found as one of the principal responsible for biomass recalcitrance in biofuel production (Batalha et al., 2015). Lignin varies between species and also between cellular types inside the same tree/plant (Campbell and Sederoff, 1996; Neutelings, 2011).

The potential of Raman imaging combined with multivariate methods (e.g., VCA) to differentiate also subtle changes in lignin composition was shown in spruce wood (Gierlinger, 2014) and in this study on a Raman map of Arabidopsis including xylem vessels and sclerenchyma fibers (Figure 3). The calculated endmember spectra proofed that the lignin in the cell corners gluing together the sclerenchyma fibers has more similarity to the lignin in the xylem vessel than in the fibers itself (EM 1). Besides also changes in the matrix have been observed depending if cells are connected in the vascular tissue (EM 1) or in the mechanical stabilizing sclerenchymatic tissue (EM 2). Especially the band at 1660 cm^−1^ changes between secondary cell walls of fibers and xylem and cell corners (Figure 3H). In comparison to the reference spectra of milled wood lignin (MWL) in Figure 2, also a higher band at 1660 cm^−1^ assigned to the aromatic ring conjugated C=C bonds and to the C=O group (of coniferaldehyde and/or sinapaldehyde units in lignin; Agarwal and Ralph, 2008; Agarwal et al., 2011) was observed in Arabidopsis compared to MWL. Here more similarity was found with the artificial polymerized DHP, which showed also a higher band at 1652 cm^−1^ band and also the band at 1132 cm^−1^, assigned to coniferaldehyde/sinapaldehyde (Agarwal and Ralph, 2008; Agarwal et al., 2011), similarly pronounced like in EM spectra of Arabidopsis. The lignin most typical for the sclerenchyma fibers (Figure 3, EM 4), showed a higher band at 1336 and 1457 cm^−1^, which were also clearly seen in MWL of beech (Figure 2, spectrum **f**) and are reported to be typically for syringyl units in lignin (Agarwal and Terashima, 2003; Sun et al., 2012). The presence of higher amounts of guaicyl lignin in vessel elements and higher proportion of syringyl in fibers has been reported for wood samples (Saito et al., 2012) and is now proofed for *Arabidopsis*. Syringyl and guaiacyl units differ in their degree of methylation of the phenylpropane units having the first one methyl group more. During evolution syringyl lignin appear first in angiosperms involving two more steps in the metabolic grid whereas guiacyl lignin is typical for conifers and derives directly from coniferyl alcohol (Eckardt, 2002). CRM gives the value of S/G ratios non-destructively (Sun et al., 2012) and assesses locally differences in xylem maturation and development.

### Revealing *In situ* changes in cuticle composition on the micron-level

The cuticle covers all aerial organs in the plant and its major characteristic, hydrophobicity, is given by the nature of its composition. Poly-hydroxy and epoxy fatty acids are crosslinked by ester bonds to cutin, which is combined with a variable, generally low amount, of waxes. The disposition of the main polymers in the epidermal cells matches the one suggested in literature (Dominguez et al., 2011) with a triangular area rich in pectin (EM 3) below the cuticle (EM 1) and a half moon formed cellulose layer (EM 4). The cellulose part was divided in two parts being the upper part a mixture of pectin and cellulose (as given by the endmembers 3 and 4) whereas the inner part toward the lumen was richer in cellulose. The presence of hemicelluloses is depicted by the low frequency band at 495 cm^−1^ (Agarwal and Ralph, 1997). Comparing the cuticle EM spectrum (Figure 4, EM 1) of Arabidopsis with the acquired reference spectra of cutin monomer and tomato cuticle showed that the main lipid bands at 1443 and within 1299–1307 cm^−1^ were present in all three. The peaks at 1063 cm^−1^ (cuticular wax) and 1712 cm^−1^ (ester bond) observed in EM 1 were only in common with the insoluble cutin reference monomer (Figure 2a; for assignments see Table 1) which indicated an esterification of the latter. Tomato cuticle and cutin had the band 1172 cm^−1^ in common (characteristic for cuticular wax and/or p-coumaric acid (see Discussion below), which was not strongly visible in Arabidopsis (low intensity). This could mean that the cuticle of Arabidopsis consists mainly of cutin and only small amounts of cuticular waxes. The typical phenolic bands (1540–1680 cm^−1^ region) showed less intensity in the cuticle of the Arabidopsis stem, whereas it was clearly seen in the spectra of both isolated cutin and native tomato cuticle. In fact, the main waxes and polyesters found in Arabidopsis are alkanes (and ketones) and dicarboxylic acids, respectively (Suh et al., 2005).

The amount and nature of the lipids and phenolics can also vary due to biotic/abiotic stresses and or tissue specialization i.e., trichomes. It has been found that the fraction of phenolics in the cutin/cuticle matrix is high in gymnosperms and appears in the form of lignin (up to ~26% of the isolated cuticle; Reina et al., 2001). However, the investigation of this feature in angiosperms remains to be done. Marks et al. (2008) found that isolated leaf trichomes of Arabidopsis had a fraction of lignin which they addressed to be in the cell wall after performing the Mäule reaction on detached trichomes. In Figure 5 the VCA of a stem trichome of Arabidopsis shows the transition from a cuticle rich in lipids near the stem (EM 1) to a cuticle rich in phenolics (EM 3) since the bands at 1600 and 1630 cm^−1^, similar to the ones in coumaric acid (Figure 2, spectrum **c** in red), are prominent in the more distant part of the trichome. Ferulic acid and p-coumaric acid have been also found covalently attached to cutin and suberin in apples, peach, pear, and tomato by mass spectrometry and gas chromatography (Riley and Kolattukudy, 1975). Furthermore, ferulic acid has been found not only in fruits but also in primary cell walls of gymnosperms by UV fluorescence microscopy and treatment with NaOH (Carnachan and Harris, 2000) and ferulate makes up to 1% of the cutin polymer (Pollard et al., 2008).

It seems then that phenolic compounds are common for both cell walls and cuticles. They are precursors of lignin and can also be incorporated in lignin of angiosperms (Ralph et al., 2008) and grasses (Lam et al., 2001). In *Brachypodium* contributions of ferulic acid have been found not only in the cell wall but also in cell corners (Gierlinger et al., 2013). We found that p-coumaric and/or ferulic acid were part of the cuticle at the hair but not of the epidermal cell wall by the presence of the peaks at 1171, 1266, and the doublet at 1606 and 1632 cm^−1^ (Piot et al., 2001; Ram et al., 2003). The extracted cutin monomer and the native tomato cuticle (Figures 2a,b spectra, respectively) were also characterized by the doublet and the band at 1171–1177 cm^−1^ indicating p-coumaric acid in these reference samples. The presence of phenolic compounds is related to an increase in the rigidity of the cutin matrix (López-Casado et al., 2007). The cutin monomer spectrum (mainly non-esterified hydroxy fatty acids) was more similar to the EM 1 in Figure 5B due to the presence of the peaks at 1064 and 1133 cm^−1^ attributed to the C–C stretching of wax and cutin matrix (Prinsloo et al., 2004; Yu et al., 2007, 2008; Trebolazabala et al., 2013).

The potential of the Raman imaging approach in studying non-polysaccharide components is clearly seen in this study by revealing a molecular fingerprint on the micro-level. By this detailed information is gained on: (1) where are the components within the plant cells and (2) what is the chemical nature of the components. If combined with VCA even subtle changes in chemical compositions can be tracked. The advantage of VCA falls on the fact that it extracts the most pure components in the pixel matrix and reveals the correspondent abundance or distribution maps.

## Author contributions

NG: research idea and experiment design, data analysis, and writing of the manuscript; BP: Raman experiments, data analysis, and writing of the manuscript; AH: preparation of cutin monomer, and tomato cuticle, scientific input regarding all aspects of cuticles in the manuscript; MH: providing the Arabidopsis sample and English corrections.

## Funding

Austrian Science Fund (FWF): START Project [Y-728-B16].

### Conflict of interest statement

The authors declare that the research was conducted in the absence of any commercial or financial relationships that could be construed as a potential conflict of interest.
